# The complete chloroplast genome of *Coffea liberica* (Gentianales: Rubiaceae)

**DOI:** 10.1080/23802359.2022.2107459

**Published:** 2022-08-08

**Authors:** Xuehui Bai, Hongyu Zheng, Xing Huang, Jinhong Li, Tieying Guo, Qin Luo, Zhirun Zhang, Weihuai Wu, Kexian Yi

**Affiliations:** a Dehong Tropical Agriculture Research Institute of Yunnan, Ruili, PR China; b China State Farms Economic Development Center, Beijing, PR China; c Environment and Plant Protection Institute, Chinese Academy of Tropical Agricultural Sciences, Haikou, PR China; d Key Laboratory of Integrated Pest Management on Tropical Crops, Ministry of Agriculture and Rural Affairs, Haikou, PR China; e Hainan Key Laboratory for Monitoring and Control of Tropical Agricultural Pests, Haikou, PR China

**Keywords:** *Coffea liberica*, chloroplast genome, phylogenetic tree

## Abstract

Coffee is one of the most popular beverages around the world. As one of the best-known coffee species, Liberian coffee (*Coffea liberica* Bull ex Hiern 1876) has a high resistance to leaf rust, a devasting disease caused by *Hemileia vastatrix*. However, there are few reports on the systematic position and phylogenetic relationship of *C. liberica* at the chloroplast (cp) genome level. Thus, we successfully assembled its cp genome. The full length is 154,799 bp with a GC content of 37.48%. We have further annotated the cp genome and predicted 85 protein-coding genes together with 8 rRNAs and 37 tRNAs. Furthermore, a large single copy region (LSC), a small single copy region (SSC), an inverted repeat region a (IRa) and an inverted repeat region b (IRb) are identified with lengths of 84,868 bp, 18,121 bp, 25,905 bp and 25,905 bp, respectively. The phylogenetic tree indicates that *C. liberica* is closely related to *C. canephora*, which is consistent with a previous result obtained from genotyping‐by‐sequencing.

## Background

Coffee is one of the most popular beverages around the world. The three best-known coffee species for coffee production are Arabica (*Coffea arabica* L.), Robusta (*C. canephora* L. Linden) and Liberian coffees (*C. liberica* Bull ex Hiern 1876) (Patay et al. 2016). To date, *C. arabica* has the largest cultivation areas for coffee production, but it is threatened by leaf rust, a devasting disease caused by *Hemileia vastatrix* (Talhinhas et al. 2017). In contrast, high leaf rust resistance has been identified in *C. canephora* and *C. liberica*, which has been successfully used for breeding resistant varieties in *C. arabica* (Prakash et al. 2004). However, there are few reports on the systematic position and phylogenetic relationship of *C. liberica* at the chloroplast (cp) genome level. The cp genome could provide reliable evidence of the evolution and origin of plant species, such as Solanaceae (Mehmood, Shahzadi, et al. 2020; Mehmood, Ubaid, Bao, et al. 2020; Mehmood, Ubaid, Shahzadi, et al. 2020). Thus, we successfully sequenced and assembled the cp genome of *C. liberica*, which will benefit related studies in the future.

## Methods and results

Young leaves of *C. liberica* were cut from a five-year-old tree in the coffee germplasm garden of the Dehong Tropical Agriculture Research Institute of Yunnan in Ruili, China (24.0256°N, 97.8596°E) and used for DNA extraction. The specimen has been preserved in the Herbarium of the Dehong Tropical Agriculture Research Institute of Yunnan (http://www.dtari.org.cn/, Xuehui Bai, 13529520059@163.com) under the voucher number DTARI-cl202101. The fresh leaves were rapidly soaked in liquid nitrogen and broken into powder for total DNA extraction by using the CTAB method (Doyle and Doyle 1987). The DNA sample was used for library construction and Illumina sequencing after being delivered to Biozeron Biotech (Shanghai, China). The Illumina NovaSeq platform was selected for paired-end short reads sequencing after the DNA sequences were broken into 300–500 bp fragments. After Illumina sequencing, we deposited a total of 3.81 Gb raw data in the SRA database with the accession number PRJNA771824. A total of 3.78 Gb clean data was filtrated in order to assemble the scaffolds of the cp genome by using NOVOPlasty v4.2 (Dierckxsens et al. 2017). The gaps between scaffolds were filled with GapCloser v1.12 to obtain the full cp genome (Luo et al. 2012). The cp genome of *C. liberica* contained 154,799 bp with a GC content of 37.48%, which was deposited in GenBank under the accession number MW970411. We selected the GeSeq and CPGAVAS2 software to annotate the cp genome and predicted 85 protein-coding genes together with 8 rRNAs and 37 tRNAs (Tillich et al. 2017; Shi et al. 2019). We selected Geneious v11.0.3 to screen the regional boundaries (Kearse et al. 2012). As a result, a large single copy region (LSC), a small single copy region (SSC), an inverted repeat region a (IRa) and an inverted repeat region b (IRb) were identified with lengths of 84,868 bp, 18,121 bp, 25,905 bp and 25,905 bp, respectively.

**Figure 1.**
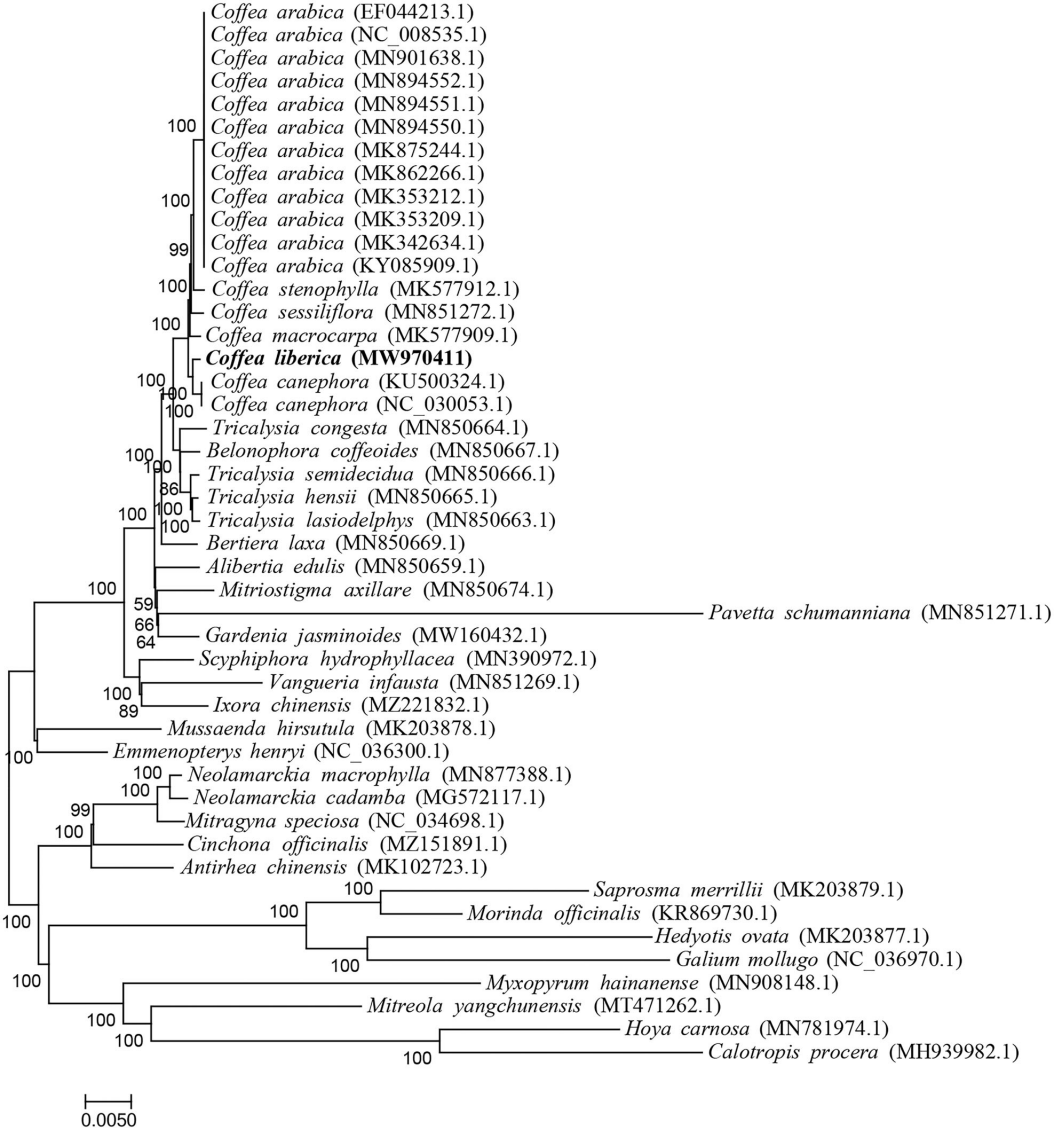
Phylogenetic tree of 46 chloroplast genomes.

We selected 46 cp genome sequences to conduct the phylogenetic analysis. There are 42 species in Rubiaceae and four other species as outgroup, comprising *Myxopyrum hainanense*, *Mitreola yangchunensis*, *Hoya carnosa* and *Calotropis procera* (Amenu et al. 2022). All these cp genomes were aligned using MAFFT v7.0 (Katoh and Standley 2013). The phylogenetic tree was constructed by the Maximum Likelihood method with bootstrap values of 1000 replicates in MEGA 7.0.26 (Kumar et al. 2016). The result has indicated that *C. liberica* is closely related to *C. canephora* (Figure 1), which is consistent with a previous result obtained from genotyping‐by‐sequencing (Bawin et al. 2021). This study will benefit future studies related to chloroplast in the *Coffea* genus.

## Data Availability

The genome sequence data that support the findings of this study are openly available in GenBank of NCBI at (https://www.ncbi.nlm.nih.gov/nuccore/) under the accession number MW970411. The accession numbers of BioProject, SRA and Bio-Sample are PRJNA771824, SRX12645655 and SAMN22346234, respectively.

## References

[CIT0001] Amenu SG, Wei N, Wu L, Oyebanji O, Hu G, Zhou Y, Wang Q. 2022. Phylogenomic and comparative analyses of Coffeeae alliance (Rubiaceae): deep insights into phylogenetic relationships and plastome evolution. BMC Plant Biol. 22(1):88. 35219317 10.1186/s12870-022-03480-5 PMC8881883

[CIT0002] Bawin Y, Ruttink T, Staelens A, Haegeman A, Stoffelen P, Mwanga JCI, Roldán-Ruiz I, Honnay O, Janssens SB. 2021. Phylogenomic analysis clarifies the evolutionary origin of *Coffea arabica*. J Syst Evol. 59(5):953–963.

[CIT0003] Dierckxsens N, Mardulyn P, Smits G. 2017. NOVOPlasty: de novo assembly of organelle genomes from whole genome data. Nucleic Acids Res. 45(4):e18. 28204566 10.1093/nar/gkw955 PMC5389512

[CIT0004] Doyle JJ, Doyle JL. 1987. A Rapid DNA isolation procedure from small quantities of fresh leaf tissues. Phytochem Bull. 19:11–15.

[CIT0005] Katoh K, Standley DM. 2013. MAFFT multiple sequence alignment software version 7: improvements in performance and usability. Mol Biol Evol. 30(4):772–780. 23329690 10.1093/molbev/mst010 PMC3603318

[CIT0006] Kearse M, Moir R, Wilson A, Stones-Havas S, Cheung M, Sturrock S, Buxton S, Cooper A, Markowitz S, Duran C, et al. 2012. GeneiousBasic: an integrated and extendable desktop software platform for the organization and analysis of sequence data. Bioinformatics. 28(12):1647–1649. 22543367 10.1093/bioinformatics/bts199 PMC3371832

[CIT0007] Kumar S, Stecher G, Tamura K. 2016. MEGA7: molecular evolutionary genetics analysis version 7.0 for bigger datasets. Mol Biol Evol. 33(7):1870–1874. 27004904 10.1093/molbev/msw054 PMC8210823

[CIT0008] Luo R, Liu B, Xie Y, Li Z, Huang W, Yuan J, He G, Chen Y, Pan Q, Liu Y, et al. 2012. Soapdenovo2: an empirically improved memory-efficient short-read de novo assembler. GigaSci. 1(1):1. 10.1186/2047-217X-1-18 PMC3626529 23587118

[CIT0009] Mehmood F, Shahzadi I, Ahmed I, Waheed MT, Mirza B. 2020. Characterization of *Withania somnifera* chloroplast genome and its comparison with other selected species of Solanaceae. Genomics. 112(2):1522–1530. 31470082 10.1016/j.ygeno.2019.08.024

[CIT0010] Mehmood F, Ubaid Z, Bao Y, Poczai P, Mirza B. 2020. Comparative plastomics of ashwagandha (Withania, Solanaceae) and identification of mutational hotspots for barcoding medicinal plants. Plants. 9(6):752. 10.3390/plants9060752 PMC7355740 32549379

[CIT0011] Mehmood F, Ubaid Z, Shahzadi I, Ahmed I, Waheed MT, Poczai P, Mirza B. 2020. Plastid genomics of *Nicotiana* (Solanaceae): insights into molecular evolution, positive selection and the origin of the maternal genome of Aztec tobacco (*Nicotiana rustica*). PeerJ. 8:e9552. 32775052 10.7717/peerj.9552 PMC7382938

[CIT0012] Patay ÉB, Bencsik T, Papp N. 2016. Phytochemical overview and medicinal importance of *Coffea* species from the past until now. Asian Pac J Trop Med. 9(12):1127–1135. 27955739 10.1016/j.apjtm.2016.11.008

[CIT0013] Prakash NS, Marques DV, Varzea VM, Silva MC, Combes MC, Lashermes P. 2004. Introgression molecular analysis of a leaf rust resistance gene from *Coffea liberica* into *C. arabica* L. Theor Appl Genet. 109(6):1311–1317. 15241596 10.1007/s00122-004-1748-z

[CIT0014] Shi L, Chen H, Jiang M, Wang L, Wu X, Huang L, Liu C. 2019. CPGAVAS2, an integrated plastome sequence annotator and analyzer. Nucleic Acids Res. 47(W1):W65–W73. 31066451 10.1093/nar/gkz345 PMC6602467

[CIT0015] Talhinhas P, Batista D, Diniz I, Vieira A, Silva DN, Loureiro A, Tavares S, Pereira AP, Azinheira HG, Guerra-Guimarães L, et al. 2017. The coffee leaf rust pathogen *Hemileia vastatrix*: one and a half centuries around the tropics. Mol Plant Pathol. 18(8):1039–1051. 27885775 10.1111/mpp.12512 PMC6638270

[CIT0016] Tillich M, Lehwark P, Pellizzer T, Ulbricht-Jones ES, Fischer A, Bock R, Greiner S. 2017. GeSeq - versatile and accurate annotation of organelle genomes. Nucleic Acids Res. 45(W1):W6–W11. 28486635 10.1093/nar/gkx391 PMC5570176

